# Mapping the Evidence on Virtual Reality for Post-Intensive Care Syndrome: A Systematic Review and a Five-Axis VR-PICS Taxonomy

**DOI:** 10.3390/biomedicines14020464

**Published:** 2026-02-19

**Authors:** Inês Oliveira, André Torneiro, João Ferreira-Coimbra, Adriana Sampaio, Nicolas A. Morgenstern, Eva Oliveira, António Coelho, Nuno F. Rodrigues

**Affiliations:** 1 Faculty of Engineering, University of Porto, 4200-465 Porto, Portugal; acoelho@fe.up.pt; 2 Logimade, 9000-216 Funchal, Portugal; andre.torneiro@logimade.pt; 3 Internal Medicine Service, Porto University Hospital Center, 4200-319 Porto, Portugal; joaoferreiracoimbra@gmail.com; 4 School of Psychology, University of Minho, 4710-057 Braga, Portugal; adriana.sampaio@psi.uminho.pt; 5 Virtuleap, 1300-501 Lisbon, Portugal; 6 Institute for Systems and Computer Engineering, Technology and Science (INESC-TEC), 4200-465 Porto, Portugal; eoliveira@ipca.pt (E.O.); nuno.f.rodrigues@inesctec.pt (N.F.R.)

**Keywords:** systematic review, virtual reality, intensive care unit, post-intensive care syndrome, rehabilitation, critical care, taxonomy

## Abstract

**Background:** Post-Intensive Care Syndrome (PICS), comprising physical, cognitive, and psychological impairments, affects 50–75% of Intensive Care Unit (ICU) survivors and leads to long-term deficits. Virtual Reality (VR) has emerged as a tool to reduce ICU-related stress and support recovery, yet evidence remains fragmented and heterogeneous. **Objective:** To systematically review the safety, feasibility, and effects of immersive VR interventions targeting PICS-related outcomes in ICU and post-ICU populations, and to introduce a standardized taxonomy to classify and compare VR interventions in critical care contexts. **Methods:** This systematic review followed PRISMA 2020 guidelines and was registered in PROSPERO (CRD420251174623). Seven databases (Cochrane Library, PubMed, ScienceDirect, IEEE Xplore, ACM Digital Library, SpringerLink, and Scopus) were searched from inception to 2 August 2025. Eligible studies included ICU patients receiving immersive VR via head-mounted displays and targeting at least one PICS domain. Two reviewers independently screened studies and extracted data. Methodological quality was assessed using the Mixed Methods Appraisal Tool (MMAT, 2018). Due to substantial heterogeneity, findings were synthesized narratively. **Results:** Eleven studies were included. The most consistent effects concerned acute psychological outcomes, with 63.6% of studies reporting reduced anxiety or distress. Evidence for physical, cognitive, or long-term outcomes was limited and inconsistent, largely due to small samples, non-randomized designs, and brief intervention dosing. **Conclusion:** Current evidence supports VR as a feasible adjunct for acute psychological support in ICU settings. However, meaningful rehabilitation effects remain underexplored. The Five-Axis VR-PICS taxonomy clarifies intervention heterogeneity and provides a structured framework to guide rehabilitation-oriented VR research in critical care.

## 1. Introduction

PICS affects an estimated 50–75% of ICU survivors [1,2,3,4,5,6,7]. Despite improvements in critical care survival, many survivors—particularly those who required mechanical ventilation—experience persistent physical, cognitive, and psychological impairments that adversely impact daily functioning, return to work, and healthcare utilization [1,2,3,7,8,9]. These deficits—ranging from ICU-acquired weakness (ICUAW) and reduced mobility to memory disturbance, slowed processing, anxiety, depression, and Post-Traumatic Stress Disorder (PTSD) [2,4,8,10,11]—are often reinforced by the ICU environment, which combines sensory overload with isolation, promoting distress, disorientation, and cognitive fatigue [4,5,6,7,10,11,12,13]. Core risk factors such as prolonged ventilation, deep sedation, and delirium remain central to prevention [2,3,5,8,10].

The ABCDEF bundle integrates delirium monitoring, early mobility, and family engagement and remains the standard for mitigating PICS [2,3,5,8]. Early mobilization improves functional outcomes and shortens ICU stays [2,3,8,14], and ICU diaries reduce PTSD [2,3], yet the high prevalence of PICS indicates the need for scalable interventions that can accompany patients across the ICU and post-ICU trajectory [3,4].

VR has emerged as a promising adjunct intervention [1,4,7,9,13,15,16]. Delivered through Head-Mounted Displays (HMDs), VR provides immersive multisensory environments [4,5,6,7,17,18] and is effective for pain, anxiety, and motor rehabilitation in diverse clinical populations [7,14,18,19,20,21]. In the ICU, VR has been reported to reduce environmental stress, lower anxiety, promote attentional relief [4,5,6,7,13,19,20,21], and facilitate participation in early physical and cognitive rehabilitation to help prevent ICUAW [1,4,14,18,20].

However, the VR-ICU literature remains early-stage and highly heterogeneous. Studies differ substantially in clinical timing, therapeutic intent, VR modality (passive vs. active), content design, dose, and outcome selection. Importantly, most interventions do not evaluate PICS prevention as a defined endpoint but rather assess proximal psychological, physical, or cognitive outcomes aligned with PICS domains across different phases of recovery. This heterogeneity limits comparability and hinders identification of which VR configurations may be relevant for rehabilitation-oriented pathways. Accordingly, this systematic review aims to map and critically appraise the available evidence on immersive HMD-based VR interventions in ICU and early post-ICU settings, focusing on safety, feasibility, and reported effects across PICS-related domains. During synthesis, a structured multi-axis taxonomy was developed and applied to make sources of heterogeneity explicit and to support clearer interpretation of the current evidence, with the goal of informing future VR research in critical care.

## 2. Materials and Methods

### 2.1. Protocol and Search Strategy

This systematic review was conducted in accordance with the PRISMA 2020 guidelines and prospectively registered in PROSPERO (CRD420251174623). We searched seven databases from inception to 2 August 2025: Cochrane Library, PubMed, ScienceDirect, IEEE, ACM, Springer Nature Link, and Scopus.

Given the variability and overlap of terminology related to virtual reality within clinical, simulation, and rehabilitation literature, a broad search strategy was adopted. The core Boolean string used across databases was: (vr OR “virtual reality”) AND (icu OR “intensive care unit”).

Searches were limited to titles, abstracts, and keywords where supported, or equivalent subject fields depending on the database structure. For SpringerLink, additional subject and language filters were applied (English language; subject areas related to virtual and augmented reality and intensive or critical care medicine) to improve specificity. No date restrictions were applied. Full database-specific search strategies are provided in Supplementary Material S1.

### 2.2. Research Questions and Objectives

This systematic review was guided by the following primary research question: What evidence exists regarding the safety, feasibility, and reported effects of immersive virtual reality (VR) interventions delivered during or after ICU admission for outcomes relevant to Post-Intensive Care Syndrome (PICS)?

The question was informed by a modified PICO framework, in which the population included ICU and post-ICU inpatients of any age, the intervention was immersive VR delivered via head-mounted displays, and the outcomes comprised psychological, physical, and cognitive measures relevant to PICS. Formal comparators were not required due to the exploratory and heterogeneous nature of the evidence base.

During synthesis, marked heterogeneity was observed across studies in terms of therapeutic intent, timing, VR modality, content, and dose. To address this limitation and support structured comparison, we developed the Five-Axis VR-PICS (5A-VR-PICS) taxonomy, derived from the characteristics of the included studies.

Accordingly, the objectives of this review were to (1) map existing evidence on immersive VR interventions relevant to PICS outcomes and (2) apply a standardized taxonomy to improve clarity, comparability, and methodological transparency, with the aim of informing future VR research in critical care.

### 2.3. Selection Process

All retrieved records were exported and duplicated prior to screening. Study selection occurred in two stages. Two reviewers (I.O. and A.T.) independently screened titles and abstracts to identify potentially eligible studies, followed by independent full-text assessment. Disagreements at any stage were resolved through discussion and consensus. No automation tools or artificial intelligence-assisted screening methods were used.

### 2.4. Exclusion and Inclusion Criteria

Eligibility criteria were defined a priori and applied consistently during screening.

Inclusion criteria were: (1) empirical studies involving ICU or post-ICU inpatient populations of any age; (2) use of immersive virtual reality delivered via a head-mounted display (HMD); (3) targeting at least one domain of Post-Intensive Care Syndrome (psychological, physical, or cognitive); (4) clear description of the VR intervention; (5) reporting of evaluation methods; and (6) presentation of outcome data or analyses relevant to the intervention.

Exclusion criteria were: (1) full-text articles unavailable; (2) publications not written in English or Portuguese; (3) non-interventional or non-empirical publications (e.g., reviews, protocols, editorials); (4) studies not primarily focused on virtual reality interventions; and (5) studies involving non-ICU populations, including healthy volunteers. Studies delivering VR exclusively after hospital discharge were also excluded.

These criteria ensured inclusion of studies with sufficient methodological and outcome detail to support structured synthesis and taxonomy-based classification.

### 2.5. Data Extraction and Charting

Data were extracted using a pre-defined, structured Google Sheet designed to capture all variables required for evidence synthesis and Five-Axis VR-PICS taxonomy classification. Data extraction was performed by one reviewer (I.O.) and independently cross-validated by a second reviewer (A.T.) to ensure accuracy and completeness. Any discrepancies were resolved through discussion and consensus.

Extracted study characteristics included study design, sample size, patient demographics, clinical context, ICU length of stay, illness severity indicators when reported, and baseline cognitive or mobility status. Intervention-related data comprised VR hardware characteristics, immersion level, interaction modality, content format, content origin, degree of customization or adaptiveness, supervising personnel, session duration, frequency, total intervention period, and logistical considerations relevant to feasibility.

Outcome data encompassed all reported psychological, cognitive, and physical measures relevant to PICS domains, including the validated instruments used (e.g., HADS, MoCA, MMI, PTSD-related scales), timing of outcome assessments, and quantitative findings as presented in the original studies. All outcomes reported within eligible studies were extracted without selective prioritization of specific timepoints or analyses. Feasibility and safety indicators—including comfort, usability, acceptability, adherence, and adverse events—were also recorded.

When information required for extraction or taxonomy classification was unclear or not reported, this was documented as not reported, and no imputation or inference was performed. Study authors were not contacted for additional data.

### 2.6. Data Synthesis

Given the substantial clinical, methodological, and intervention heterogeneity across included studies, a narrative synthesis was conducted. No meta-analysis or quantitative pooling was attempted. Formal certainty-of-evidence assessment was not performed, as the primary aim of the review was evidence mapping and intervention classification rather than effect estimation.

Studies were grouped and synthesized according to: (1) PICS domain(s) addressed (psychological, physical, cognitive) and (2) key intervention characteristics as structured by the Five-Axis VR-PICS taxonomy.

All included studies were coded using the 5A-VR-PICS taxonomy by one reviewer (I.O.) and independently cross-validated by a second reviewer (N.F.R.). This process enabled structured comparison of interventions with similar therapeutic intent but differing design features and highlighted clusters, under-reported variables, and methodological gaps.

### 2.7. Critical Appraisal and Risk of Bias

Methodological quality and risk of bias were independently assessed by two reviewers (I.O. and A.T.) using the Mixed Methods Appraisal Tool (MMAT, 2018), which is appropriate for reviews incorporating randomized, non-randomized, observational, feasibility, and mixed-methods studies. Disagreements were resolved through discussion and consensus.

Consistent with MMAT guidance, no numerical quality score was calculated. Instead, domain-level assessments were used to assign an overall judgment of Low or High Risk of Bias based on critical methodological limitations.

Due to the limited number of included studies and the absence of comparable effect estimates, formal assessment of publication bias (e.g., funnel plots) was not conducted. Detailed study-level assessments are provided in Supplementary Table S2.

## 3. Results

### 3.1. Search Outcome

The literature search identified 734 records. After removal of 174 duplicates, 560 records were screened based on titles and abstracts, of which 517 were excluded for not meeting the eligibility criteria. Forty-three full-text reports were assessed for eligibility, and 32 were excluded based on predefined inclusion and exclusion criteria, including use of non-HMD VR systems, non-ICU populations, or insufficient methodological or outcome data. Eleven studies met all inclusion criteria and were included in the final synthesis.

**Figure 1:**
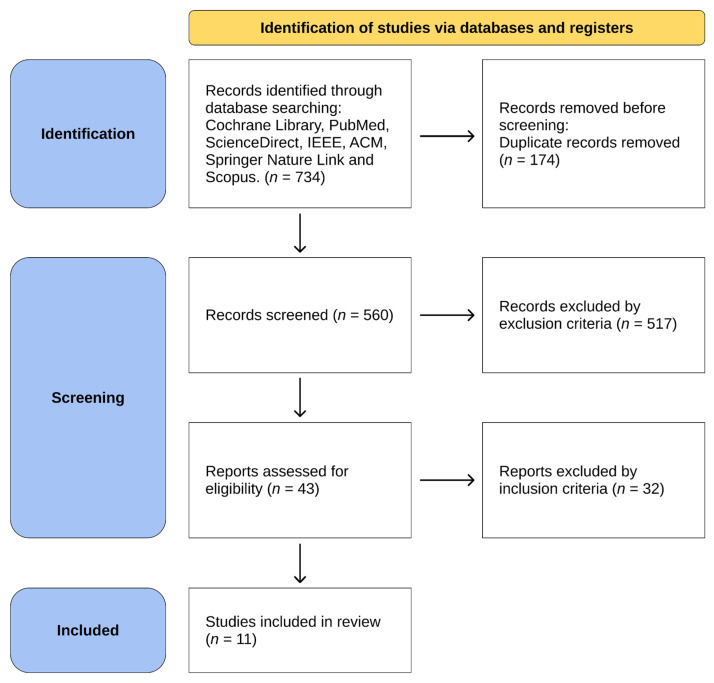
PRISMA flow diagram for study selection. *n*: Number.

The study selection process is illustrated in the PRISMA 2020 flow diagram (Figure 1). A detailed list of full-text reports excluded after eligibility assessment, with reasons for exclusion, is provided in Supplementary Table S3.

### 3.2. Overview of Included Studies

The 11 included studies represent an early-stage but thematically coherent body of evidence evaluating immersive, HMD-based VR interventions delivered during ICU admission or early inpatient recovery phases prior to hospital discharge. Due to substantial heterogeneity in study design, clinical context, and therapeutic intent, results are presented using a structured descriptive approach.

Study design, population type, clinical phase, and primary therapeutic aim are summarized in Table 1, while detailed demographic characteristics and care settings are provided in Supplementary Table S4.

#### 3.2.1. Study Design and Methodological Features

Among the 11 included studies, 5 (45.5%) were feasibility studies [1,7,11,13,16], 3 (27.3%) were RCTs [9,12,15], 2 (18.2%) were case reports [10,14], and 1 (9.1%) was observational [20]. Ten studies used prospective designs [1,7,9,10,11,12,13,14,15,16], and one was cross-sectional [20]. Quantitative methods were used in 9 studies [1,7,9,10,11,12,15,16,20], while 2 used mixed methods [13,14].

Common safety-related exclusion criteria included severe visual/hearing impairment [5,7,15,16,21], delirium [1,7,12,15,16], neurological or psychiatric disorders [1,7,11,15,16], and cases requiring external oxygen support [7,12,15,21].

#### 3.2.2. Patient Demographics and Intervention Settings

Ten studies enrolled adults (mean/median ages 55.4–68.7 years) [1,7,9,10,11,12,13,15,16], and one enrolled pediatric patients (mean age 12.9 years) [20]. Adult cohorts were predominantly male (55–100%). Clinical populations included general ICU patients (4/11; 36.4%) [7,11,13,16], cardiac surgery patients (3/11; 27.3%) [11,12,15], COVID-19 survivors (2/11; 18.2%) [9,10], and pediatric patients (2/11; 18.2%) [14,20].

Six studies (54.5%) delivered VR during the acute ICU stay [7,11,12,13,15,16], and five (45.5%) delivered VR in post-ICU inpatient rehabilitation or postoperative settings [1,9,10,14,20]. Seven studies were single-center [10,11,12,14,15,16,20], and four were multicenter [1,7,9,13].

### 3.3. VR Interventions

Across the 11 included studies, immersive VR interventions varied substantially in hardware configuration, interaction modality, content type, and therapeutic dose. VR interventions were characterized along six core dimensions: HMD model, interaction paradigm (passive vs. active), content category, content type, dose (session duration, frequency, and total exposure), and delivering personnel. Table 2 provides a structured overview of these intervention characteristics.

#### 3.3.1. Hardware, Interaction, and Content Characteristics

Most interventions were delivered using untethered head-mounted displays (6/11; 54.5%) [1,7,9,12,13,16], facilitating bedside use in ICU settings. Passive interaction paradigms predominated, with 9 of 11 studies (81.8%) relying exclusively on non-interactive VR experiences in which patients acted as observers rather than active participants [6,7,9,10,12,13,15,16,20]. Only three studies (27.3%) incorporated active VR, requiring motor or controller-based interaction, and these were exclusively used for physical or cognitive rehabilitation purposes [1,14,21].

##### Passive VR Content

Content delivery was dominated by 360° videos (7/11; 63.6%) [6,7,9,10,13,16,20], most commonly designed for psychological relaxation (6/11; 54.5%) [6,7,12,13,16,20]. These environments typically depicted synthetic or natural scenes, including virtual landscapes, underwater environments, and mountain cabins [12,13], as well as broader natural settings (3/11; 27.3%) [7,13,20]. Less frequently, content included urban travel experiences [13], aquatic worlds [6], or animal-based scenes [20] (each 1/11; 9.1%).

Two studies (18.2%) implemented ICU-orientation VR, designed to familiarize patients with ICU equipment, procedures, and surroundings [9,10]. In these interventions, 360° videos incorporated explanatory voice-overs addressing mechanical ventilation, monitoring devices, and the ICU environment, aiming to reduce anxiety and improve contextual understanding during or shortly after critical illness.

Additional passive formats included simulation-based VR (2/11; 18.2%) [12,15], typically combining immersive environments with guided breathing or body awareness exercises, and animated narrative content (2/11; 18.2%) [7,16], sometimes used to enhance engagement or emotional regulation.

##### Active and Game-Based VR Content

Game-based VR was implemented in three studies (27.3%) [1,14,16], all of which targeted physical or cognitive activation rather than psychological relaxation. Two studies (18.2%) adapted commercial exergaming titles, such as Beat Saber and Thrill of the Fight, to support early mobilization or exercise engagement [14,21]. One study (9.1%) employed a research-specific puzzle-based VR system designed to stimulate motor control and coordination through task-oriented interaction [1].

Active VR interventions were consistently delivered during sub-acute or rehabilitation phases, involved longer session durations, and required supervision by physical therapists, reflecting higher patient engagement demands compared with passive ICU-based protocols.

##### Adaptiveness and Personalization

Most VR interventions were non-adaptive (7/11; 63.6%) [7,9,10,11,12,15,16], delivering fixed content without real-time adjustment to patient performance or clinical status. Limited customization—primarily allowing patients to select preferred environments—was reported in four studies (18.2%) [13,20]. Only one study (9.1%) tailored VR content based on individual physical or cognitive capacity [14], and one study (9.1%) incorporated difficulty scaling driven by motor performance metrics [1].

#### 3.3.2. Personnel, Dosage, Logistics, and Safety

Personnel varied by clinical intent: ICU nurses administered VR in psychological-focused RCTs [9,12], while physical therapists supervised rehabilitation-oriented VR [1,14]. Other studies were managed by research staff [11,13,16,20].

Session duration ranged from 5 to 10 min for brief relaxation protocols [6] to 35 min for active rehabilitation [14]. Session frequency varied from single exposures [13,20] to structured multi-week programs, such as five sessions per week for four weeks [1].

Operational challenges were reported in larger studies. El Mathari et al. (2024) documented high refusal rates, with 24/96 patients discontinuing due to insufficient emotional or cognitive readiness [15]. De Vries et al. (2025) noted that only 57% of total therapy time involved active VR because of setup and troubleshooting requirements [1].

### 3.4. Outcomes and Evaluation

Outcomes were analyzed and reported according to the three core domains of Post-Intensive Care Syndrome (PICS): psychological (PICS-P), physical (PICS-F), and cognitive (PICS-C). Given the heterogeneity of outcome measures, assessment timepoints, and study designs, results were synthesized descriptively and structured by domain.

Overall, psychological outcomes were the most frequently evaluated, followed by physical outcomes, while cognitive outcomes were assessed less consistently. To improve clarity and reduce table complexity, outcome reporting is presented in four steps: (1) an overview of outcome domains and timing (Table 3), followed by (2) domain-specific synthesis for psychological (Table 4), (3) physical (Table 5), and (4) cognitive outcomes (Table 6).

#### 3.4.1. Overview of Outcome Domains and Assessment Timing

Across the 11 included studies, anxiety was the most frequently assessed outcome (7/11; 63.6%) [7,9,10,12,13,15,16], followed by depression (4/11; 36.4%) [7,9,10,16]. Physiological stress markers—including heart rate (HR), respiratory rate (RR), blood pressure (BP), and oxygen saturation—were evaluated in four studies (36.4%) [7,11,12,13]. Post-traumatic stress disorder (PTSD) [9,10] and delirium [7,16] were each assessed in two studies (18.2%). Quality of life outcomes were reported in two studies (18.2%) [9,15].

Physical outcomes were exclusively evaluated in studies using active VR, focusing on range of motion (ROM), mobility, strength, and upper-extremity motor function [1,14].

Cognitive outcomes included delirium screening, content recollection, and exploratory cognitive testing but were inconsistently assessed and rarely followed longitudinally [7,11,16].

#### 3.4.2. Psychological Outcomes (PICS-P)

Psychological outcomes were reported in 9 of 11 studies (81.8%) [7,9,10,11,12,13,15,16]. Acute anxiety reduction was the most consistent effect. Locke et al. (2024) [13] reported significant reductions in VAMS anxiety and NVAAS stress after a single VR session, with stable HR, RR, and BP. Rousseaux et al. (2022) [12] similarly found lower anxiety VAS scores after VR, without changes in HR, AP, RR, pupil size, or OS. Ong et al. (2020) [7] observed reduced HADS-A with stable vitals.

Gerber et al. (2019) [11] reported a significant RR reduction (−1.88 breaths/min; *p* < 0.05). Badke et al. (2022) [20] found 89% of children rated VR as calming, although HRV showed no autonomic change. The largest RCT (el Mathari et al., 2024 [15]) showed lower STAI-6 anxiety on Day 3 (median 6.0 vs. 8.0; *p* = 0.01) and a trend toward improved QoR-15 (*p* = 0.06).

Long-term findings were mixed. Vlake et al. (2022) [9] found no significant differences in IES-R, SF-36, or EQ-5D at 3–6 months. A case report (Vlake et al., 2021) [10] showed normalization of HADS and IES-R at 6 months. Suvajdzic et al. (2019) [16] observed no change in HADS-D/A, HR, or BR.

#### 3.4.3. Physical Outcomes (PICS-F)

Physical improvement occurred only with active VR. de Vries et al. (2025) [1] reported significant Morton Mobility Index (MMI) gains (*p* = 0.005) with no change in hand-grip strength and consistently low VAS pain. Lai et al. (2021) [14] observed ROM increases of 32° and 50°. Across studies, HR, OS, and RPE remained stable or within light-to-moderate exertion.

#### 3.4.4. Cognitive Outcomes (PICS-C)

Gerber et al. (2019) [11] found VR served as a cognitive stimulus, with 84.8% recollecting content via eye-tracking, but no long-term cognitive change (MoCA, EQ-5D). Eye-tracking showed no effect on visual processing. Ong et al. (2020) [7] and Suvajdzic et al. (2019) [16] reported no delirium during or after VR (CAM-ICU), indicating good cognitive safety.

## 4. The Five-Axis VR-PICS Classification (5A-VR-PICS)

VR interventions applied in ICU and post-ICU settings are highly heterogeneous and frequently described using broad, non-specific labels (e.g., “relaxation VR,” “VR therapy”). Such terminology obscures clinically relevant differences in therapeutic intent, timing, technological configuration, and intervention dose, limiting cross-study comparability and hindering identification of which VR components may drive specific benefits.

**Figure 2:**
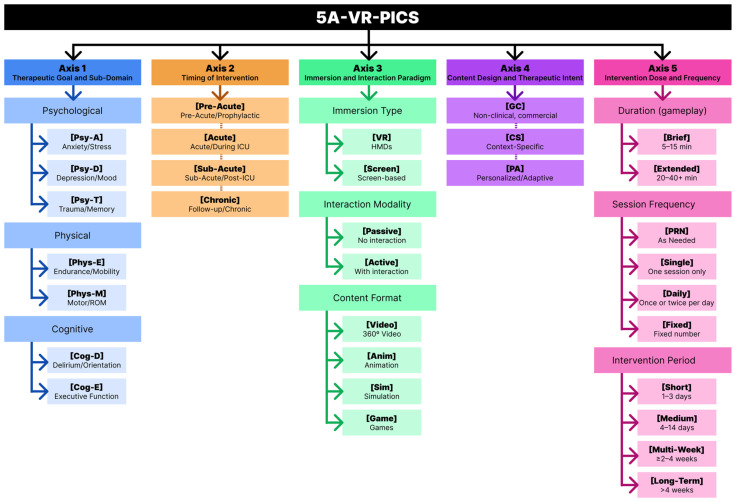
Diagram for the 5A-VR-PICS Taxonomy.

To address this limitation, we developed the Five-Axis VR-PICS Classification (5A-VR-PICS) (Figure 2), a structured, forward-looking coding framework intended for prospective use in VR studies targeting ICU and post-ICU populations. The taxonomy is designed to: (1) standardize reporting of VR interventions; (2) align intervention characteristics with targeted PICS domains and outcomes; and (3) support cumulative evidence synthesis across heterogeneous study designs.

The taxonomy comprises five complementary axes, each capturing a distinct and clinically meaningful dimension of VR intervention design. Each axis is described below, together with its corresponding classification table.

### 4.1. Axis 1: Therapeutic Goal and Sub-Domain (Clinical Target)

Many studies describe broad objectives (“reduce anxiety,” “support rehab”) without specifying the PICS sub-domain or aligning outcomes with the intended target. Axis 1 (Table 7) resolves this by dividing PICS-P (psychological), PICS-F (physical), and PICS-C (cognitive) into explicit sub-domains (e.g., anxiety, trauma memory, motor control, endurance, executive attention). This provides clearer clinical intent, enables domain-specific synthesis, supports multimodal coding, and forms the conceptual foundation for comparing VR designs and outcomes.

### 4.2. Axis 2: Timing of Intervention (Clinical Placement)

The timing of VR delivery influences feasibility, tolerability, and mechanism. Acute-phase patients typically tolerate only brief passive VR, whereas sub-acute or rehabilitation-phase patients can engage in longer, task-based VR. Many studies failed to specify timing beyond “ICU patients received VR.” Axis 2 (Table 8) categorizes interventions as Pre-Acute, Acute, Sub-Acute, or Chronic, revealing clear patterns: passive VR predominates in Acute ICU settings, whereas active VR appears mainly in Sub-Acute rehabilitation. This improves interpretability and supports development of phase-specific guidelines.

### 4.3. Axis 3: Immersion and Interaction Paradigm

Axis 3 standardizes the technological description of VR by distinguishing immersion level, interaction mode, and content structure. Because “VR” can refer to fully immersive HMDs or simple screen-based systems, separating these elements is essential. This axis enables mechanism-driven comparisons—for example, whether active controller- or hand-tracked VR provides greater motor or cognitive benefit than passive 360° viewing, or whether simulated ICU-relevant content offers advantages over generic relaxation scenes. Thus, Axis 3 (Table 9) functions as both a reporting standard and a framework for evaluating how specific technical configurations produce therapeutic effects.

### 4.4. Axis 4: Content Design and Therapeutic Intent

Axis 4 (Table 10) captures the rationale behind the VR content: (1) general purpose/commercial; (2) ICU-specific/clinically tailored; or (3) adaptive/personalized based on patient performance or status. Most included studies used non-specific commercial relaxation content, which, while engaging, does not target ICU-specific distress or cognitive/physical deficits. ICU-oriented modules (e.g., equipment explanations) and adaptive VR were rare. Making this design logic explicit supports more rigorous comparison of therapeutic mechanisms and distinguishes targeted clinical tools from generic distraction VR.

### 4.5. Axis 5: Dose and Frequency

Dose—session duration, session frequency, and total intervention period—is a critical determinant of therapeutic effect yet is the least consistently reported element across VR-ICU research. Most studies used brief, single exposures (≤15 min, once or PRN), which reliably reduce acute anxiety but are insufficient for sustained psychological, cognitive, or physical change. Axis 5 (Table 11) provides a clear structure (Duration, Frequency, Intervention Period) enabling explicit reporting and future dose–response analysis. Extended, repeated programs—aligned with rehabilitation and motor-learning principles—were rare but showed the strongest functional gains.

### 4.6. Application of the Taxonomy

#### 4.6.1. Classification of Included Studies

To demonstrate the practical applicability of the 5A-VR-PICS taxonomy and ensure transparency in how it was operationalized, the framework was retrospectively applied to all 11 studies included in this review. This application serves a dual purpose: (1) to illustrate how heterogeneous VR interventions can be systematically classified using a standardized structure, and (2) to provide a concrete example of how the taxonomy may be applied prospectively in future VR-ICU studies.

Each study was coded across all five axes based on the reported therapeutic intent, clinical phase of delivery, VR modality and interaction paradigm, content design logic, and dose parameters. When studies pursued multimodal objectives or spanned more than one clinical phase, multi-coding within an axis was permitted to reflect clinical and methodological complexity, consistent with real-world ICU practice. Unreported or insufficiently described elements were explicitly coded as [NR], highlighting persistent reporting gaps in the literature.

The retrospective application revealed clear and clinically meaningful patterns. The majority of interventions clustered around [Psy-A][Acute][VR/Passive/Video][GC][Brief/PRN], corresponding to short-term anxiety and distress reduction during ICU admission. In contrast, studies targeting physical rehabilitation consistently employed active, game-based VR, delivered in sub-acute settings with longer and more structured dosing schedules, and were associated with improvements in mobility or motor function. Cognitive-focused and adaptive VR interventions were notably scarce.

A complete classification of all included studies using the 5A-VR-PICS framework is provided in Supplementary Figure S1.

#### 4.6.2. Guidance for Application in Future Studies

Beyond its retrospective application, the 5A-VR-PICS taxonomy is intended as a prospective design and reporting tool. Future studies may apply the framework by explicitly defining each axis during intervention development and reporting, following a structured sequence:

1. Specify the targeted PICS domain(s) and sub-domain(s) (Axis 1) and align them with prespecified outcomes.
2. Define the clinical phase and care context (Axis 2) in which VR is delivered.
3. Report immersion level, interaction modality, and content format (Axis 3) to avoid ambiguity around the term “VR.”
4. Clarify content design intent (Axis 4), distinguishing generic distraction from context-specific or adaptive therapeutic content.
5. Explicitly report dose parameters (Axis 5), including session duration, frequency, and total intervention period.

Using this structure, interventions can be succinctly summarized as

Intervention = [Axis 1][Axis 2][Axis 3][Axis 4][Axis 5]

This standardized representation supports hypothesis-driven design, improves reproducibility, and enables cross-study comparison even in the presence of heterogeneous protocols.

Although the taxonomy does not prescribe optimal interventions or imply clinical effectiveness, its use may help reduce conceptual ambiguity, expose underexplored design spaces (e.g., adaptive cognitive VR), and support cumulative evidence generation in VR-based PICS rehabilitation research.

## 5. Discussion

This systematic review mapped the available evidence on immersive, HMD-based VR interventions delivered during ICU admission or early post-ICU inpatient recovery. Overall, the findings indicate that VR is feasible and generally safe in critically ill and early recovery populations, including mechanically ventilated patients. However, the evidence base remains early-stage and methodologically heterogeneous, substantially limiting conclusions regarding efficacy beyond short-term psychological outcomes.

The primary barrier to synthesis is intervention heterogeneity. The taxonomy makes this explicit: 45.5% of studies were feasibility designs, and only 27.3% were RCTs, reflecting an immature, high-risk-of-bias evidence landscape. The clearest divergence occurred in Axis 3 and Axis 4. Over 70% of studies targeted psychological outcomes and relied on passive VR (mainly 360° videos or animations), typically coded as [Psy-A][Acute][VR/Passive/Video or Anim]. Fewer than 30% addressed physical or cognitive outcomes, and these exclusively used active, game-based VR delivered at higher doses with occasional tailoring. This functional split helps explain consistent acute psychological effects versus inconsistent functional or long-term gains.

Across studies evaluating anxiety or distress in the acute ICU phase, passive VR produced reliable short-term reductions. Locke et al. (2024) [13] showed significant decreases in VAMS anxiety and stress indices with stable vital signs; Rousseaux et al. (2022) [12] found similar VAS improvements without physiological change. Gerber et al. (2019) [11] reported corroborating physiological responses—a reduction in respiratory rate (21→19 breaths/min) and systolic BP (*p* = 0.003). Together, these findings support passive VR as a non-pharmacological anxiolytic that uses sensory immersion and attentional diversion without compromising stability.

Long-term outcomes were inconsistent. Most interventions used very brief, single-session protocols (5–15 min; [Brief/Fixed] or [Brief/PRN]), insufficient to influence broader neuropsychological or functional trajectories. This aligns with Axis 5: the field is dominated by minimal dosing. The contrast between Vlake et al. (2022) [9]—showing no PTSD difference at six months after one VR session—and their case report showing improvements after two ICU-specific sessions illustrates how inadequate dose undermines therapeutic potential.

Physical and cognitive outcomes were assessed far less often, yet these domains showed the clearest rehabilitative promise. All studies evaluating physical outcomes used active VR with motor engagement. de Vries et al. (2025) [1] demonstrated substantial mobility gains in the sub-acute phase (MMI 26→57; *p* = 0.005), without changes in hand-grip strength, suggesting improvements in endurance and functional mobility. Lai et al. (2021) [14] similarly reported increased ROM with stable physiological indicators. These findings indicate that task-oriented, active VR may support PICS-F and potentially PICS-C recovery, although evidence remains limited.

Several limitations must be acknowledged. The evidence base is dominated by small feasibility studies, with only three RCTs identified, and risk of bias remains high due to non-randomized designs, small samples, and the impossibility of blinding immersive VR interventions. Reporting quality was variable, particularly for intervention content origin, adaptiveness, and dosing parameters, requiring interpretive coding in some cases. Additionally, many studies excluded patients with delirium, neurological impairment, or severe instability, biasing samples toward more stable ICU survivors and limiting generalizability.

Despite these limitations, the findings of this review have clear implications. Passive VR appears appropriate for acute psychological symptom relief in ICU settings, while active, higher-dose VR shows promise for physical rehabilitation in sub-acute phases. However, low-dose, non-specific VR should not be expected to influence long-term psychological, cognitive, or functional outcomes.

The 5A-VR-PICS framework offers a structured approach to designing, reporting, and comparing future interventions, supporting hypothesis-driven study design, dose optimization, and cumulative evidence synthesis. Future trials should explicitly justify axis-level design choices and align outcomes with therapeutic intent to advance VR from feasibility toward evidence-based ICU rehabilitation.

## 6. Designing and Reporting Future VR–PICS Studies: A Practical Guide Using the 5A-VR-PICS Framework

Evidence from this review indicates that immersive VR is safe and feasible in ICU and early post-ICU populations, with consistent short-term psychological benefits but limited evidence for sustained physical or cognitive recovery. This pattern reflects misalignment between therapeutic intent, intervention design, dose, timing, and study methodology rather than lack of therapeutic potential. Based on these findings, future VR–PICS studies should consider the following recommendations.

### 6.1. Target Explicit and Multidomain PICS Outcomes (Axis 1)

Because PICS is multidomain and most studies targeted only acute anxiety, future interventions should predefine one or more PICS sub-domains (e.g., [Psy-A], [Phys-M], [Phys-E], [Cog-E]) and allow multidomain targeting. Combining calming VR with functional or cognitive tasks (e.g., [Psy-A + Phys-M]) may better reflect real recovery trajectories and should be evaluated using domain-specific outcomes.

### 6.2. Align Intervention Timing with Recovery Phase (Axis 2)

As most acute ICU studies relied on brief passive VR, while functional gains were observed only in sub-acute rehabilitation settings, future protocols should match VR modality and intensity to patient capacity. Sequential approaches—passive VR in the acute phase followed by active VR during rehabilitation—should be explored.

### 6.3. Prefer Active and Adaptive VR for Rehabilitation (Axes 3 and 4)

Because functional improvements were observed only in studies using active VR, rehabilitation-oriented interventions should prioritize [VR/Active/Game] designs with motor and/or cognitive engagement. Given the near absence of adaptive VR, future studies should incorporate performance-based progression and task personalization, particularly for [Phys-M], [Phys-E], and [Cog-E] targets.

### 6.4. Prespecify Adequate Dose Using Rehabilitation Principles (Axis 5)

Since brief single-session VR reliably reduces anxiety but does not produce sustained effects, future studies should adopt extended sessions, fixed multi-session schedules, and multi-week intervention periods when rehabilitation is the goal. Dose parameters (duration, frequency, total period) should be explicitly reported using Axis 5 codes.

### 6.5. Choose Study Designs Appropriate to Intervention Maturity

Given that many existing RCTs are underpowered and constrained by ICU workflows, alternative designs such as cluster-randomized, stepped-wedge, or prospective cohort studies may be more suitable in early-phase research. Regardless of design, studies should compare passive versus active/adaptive VR and include objective motor, cognitive, and physiological endpoints.

### 6.6. Design VR as a Longitudinal Rehabilitation Pathway

Because PICS evolves over months, VR should be conceptualized as a longitudinal intervention spanning ICU, inpatient rehabilitation, and home recovery, with digital adherence tracking and follow-up beyond discharge (≥3–12 months).

### 6.7. Standardize Reporting Using the 5A-VR-PICS Taxonomy

As inconsistent reporting remains a major barrier to synthesis, future studies should apply the 5A-VR-PICS taxonomy prospectively to justify intervention design choices and improve transparency, comparability, and cumulative evidence generation.

## 7. Conclusions

This systematic review shows that immersive VR is safe and feasible in ICU and early post-ICU populations and is consistently associated with short-term reductions in psychological distress, particularly anxiety. Evidence for sustained physical or cognitive rehabilitation effects remains limited, largely due to small samples, heterogeneous designs, brief interventions, and inadequate dosing. Application of the Five-Axis VR-PICS taxonomy clarifies this evidence landscape, highlighting the predominance of passive, anxiety-focused VR delivered during the acute ICU phase and the scarcity of structured, rehabilitation-oriented and adaptive interventions. While VR appears well-suited as an adjunct for acute psychological support, its broader rehabilitative role in PICS remains insufficiently tested. The 5A-VR-PICS taxonomy provides a structured foundation to support more rigorous, targeted, and multidomain VR trials in critical care rehabilitation.

## Figures and Tables

**Table 1:** Characteristics of Included Studies and Patient Demographics. RCT: Randomized Controlled Trial.

Study	Design	Sample Size	Population Type	Clinical Phase	Primary Aim
Vlake et al. (2022) [9]	RCT	40/40	Adult ICU (COVID-19)	Acute ICU	Psychological (Anxiety/Stress)
Locke et al. (2024) [13]	Feasibility	20	Adult ICU	Acute ICU	Psychological (Anxiety)
Vlake et al. (2021) [10]	Case report	1	Adult ICU (COVID-19)	Acute ICU	Psychological (Orientation/Anxiety)
Ong et al. (2020) [7]	Feasibility	15	Adult ICU	Acute ICU	Psychological/Cognitive
Lai et al. (2021) [14]	Case report	2	Pediatric post-op	Sub-acute	Physical (ROM/Mobility)
Rousseaux et al. (2022) [12]	RCT	25/25	Adult cardiac surgery	Acute ICU	Psychological (Anxiety)
Badke et al. (2022) [20]	Observational	106	Pediatric ICU	Sub-acute	Psychological (Relaxation)
Gerber et al. (2019) [11]	Feasibility	35	Adult ICU	Acute ICU	Psychological/Cognitive
Suvajdzic et al. (2019) [16]	Feasibility	12	Adult ICU	Acute ICU	Psychological/Cognitive
de Vries et al. (2025) [1]	Feasibility	16	Adult post-ICU rehab	Sub-acute	Physical (Motor/Mobility)
el Mathari et al. (2024) [15]	RCT	25/25	Adult ICU	Acute ICU	Psychological (Anxiety/QoL)

**Table 2:** VR Intervention Summary. NI: Not Indicated.

Study	HMD Model	Interaction	Content Category	Content Type	Dose	Delivered by
Vlake et al. (2022) [9]	Oculus Go (Untethered)	Passive	360° videos and animations	ICU-specific 360° orientation	15–20 min; 5 sessions/1 week	ICU nurse
Locke et al. (2024) [13]	Meta Quest Pro (Untethered)	Passive	360° videos	Commercial 360° relaxation/travel	10 min; single session	Research staff
Vlake et al. (2021) [10]	NI	Passive	360° videos and animations	ICU-specific 360° orientation	20 min; 3 sessions/5 days	ICU nurse
Ong et al. (2020) [7]	Google Daydream (Untethered)	Passive	360° videos and animations	Commercial 360° relaxation/animations	10–15 min; daily up to 1 week	Study staff
Lai et al. (2021) [14]	Oculus Rift (Tethered)	Active (Hand-held controllers)	Games	Commercial exergaming	35 min; daily × 2 weeks	Physical therapist
Rousseaux et al. (2022) [12]	Oncomfort (Untethered)	Passive	Simulation	Guided simulation + breathing	15 min; 3 sessions/3 days	ICU nurse
Badke et al. (2022) [20]	NI	Passive	360° videos	Custom 360° “field trips”	10–15 min; single session	Study staff
Gerber et al. (2019) [11]	HTC Vive (Tethered)	Passive	360° videos	Custom 360° environments (+ ICU tour)	5–10 min; daily × 1 week	Research staff
Suvajdzic et al. (2019) [16]	Google Daydream (Untethered)	Passive	360° videos and animations	Commercial relaxation/animations	15 min; daily × 5 days	Research staff
de Vries et al. (2025) [1]	Oculus Quest 2 (Untethered)	Active (Hand-tracking)	Games	Research-specific adaptive puzzle VR	30 min; 5×/week × 4 weeks	Physical therapist
el Mathari et al. (2024) [15]	NI	Passive	Simulation	Research-specific breathing/body scan	10 min; daily × 3 days	Study staff

**Table 3:** Overview of Outcome Domains and Assessment Timepoints.

Study	PICS Domain Assessed	Specific Outcomes	Assessment Timepoints
Vlake et al. (2022) [9]	Psychological	Anxiety, Depression, PTSD, QoL	3, 4, 6 months post-discharge
Locke et al. (2024) [13]	Psychological	Anxiety	Pre-/post-session
Vlake et al. (2021) [10]	Psychological	Anxiety, Depression, PTSD	3–6 months post-discharge
Ong et al. (2020) [7]	Psychological, Cognitive	Anxiety, Depression, Delirium	Pre-/post-session; ≤12 h
Lai et al. (2021) [14]	Physical	ROM, Mobility	During and post-session
Rousseaux et al. (2022) [12]	Psychological	Anxiety, Physiological	Pre-/post-session
Badke et al. (2022) [20]	Psychological	Relaxation, Physiological	Pre-/during/post-VR
Gerber et al. (2019) [11]	Psychological, Cognitive	Physiological, Recollection	ICU + 3-month follow-up
Suvajdzic et al. (2019) [16]	Psychological, Cognitive	Anxiety, Delirium	Pre-/post-session
de Vries et al. (2025) [1]	Physical	Upper-extremity motor function	Baseline + follow-up
el Mathari et al. (2024) [15]	Psychological	Anxiety, QoL	Days 1–3 post-op

**Table 4:** Psychological Outcomes (PICS-P). HADS: Hospital Anxiety and Depression Scale. IES-R: Impact of Event Scale-Revised. SF-36: Short Form Health Survey (36 items). EQ-5D: EuroQol 5-Dimension (Quality of Life). VAMS: Visual Analog Mood Scale. NVAAS: Numeric Verbal Anxiety and Agitation Scale. HR: Heart Rate. RR: Respiratory Rate. BP: Blood Pressure. VAS: Visual Analog Scale (for Anxiety/Pain). HRV: Heart Rate Variability. STAI-6: State-Trait Anxiety Inventory (6-item version). QoR-15: Quality of Recovery (15-item).

Study	Evaluation Methods	Key Findings	Adverse Events
Vlake et al. (2022) [9]	HADS, IES-R, SF-36, EQ-5D	No long-term differences vs. control	None
Locke et al. (2024) [13]	VAMS, NVAAS, vitals	Significant anxiety reduction	None
Vlake et al. (2021) [10]	HADS, IES-R	Scores normalized at 6 months	None
Ong et al. (2020) [7]	HADS, vitals, CAM-ICU	Reduced anxiety/depression	One emotional reaction
Rousseaux et al. (2022) [12]	VAS, HR, RR, BP	Lower anxiety vs. control	None
Badke et al. (2022) [20]	Likert, HRV	89% reported calming effect	None
Gerber et al. (2019) [11]	HR, RR, EQ-5D	RR reduction; QoL unchanged	Mild nausea
Suvajdzic et al. (2019) [16]	HADS, IES	No significant changes	None
el Mathari et al. (2024) [15]	STAI-6, QoR-15	Lower anxiety trajectory	Nausea/discomfort

**Table 5:** Physical Outcomes (PICS-F). ROM: Range of Motion. HR: Heart Rate. OS: Oxygen Saturation. RPE: Rating of Perceived Exertion. MMI: Morton Mobility Index. VAS: Visual Analog Scale (for Anxiety/Pain).

Study	Evaluation Methods	Key Findings	Adverse Events
Lai et al. (2021) [14]	ROM, HR, OS, RPE	+32° and +50° ROM gains	Mild motion sickness
de Vries et al. (2025) [1]	MMI, grip strength, VAS	Significant MMI improvement (*p* = 0.005)	None

**Table 6:** Cognitive Outcomes (PICS-C). CAM-ICU: Confusion Assessment Method for the ICU. HADS: Hospital Anxiety and Depression Scale. MoCA: Montreal Cognitive Assessment.

Study	Evaluation Methods	Key Findings	Adverse Events
Ong et al. (2020) [7]	CAM-ICU, HADS	No delirium observed	One emotional reaction
Gerber et al. (2019) [11]	CAM-ICU, MoCA, eye-tracking	84.8% content recollection; no long-term change	Mild nausea
Suvajdzic et al. (2019) [16]	CAM-ICU	No delirium	None

**Table 7:** Axis 1—Therapeutic Goal and Clinical Target.

Domain	Sub-Domain	Code in Axis 1	Target Clinical Outcome
Psychological	Anxiety/Stress	[Psy-A]	Acute distress, agitation, or pain-related anxiety
	Depression/Mood	[Psy-D]	Depressive or prolonged emotional distress symptoms
	Trauma/Memory	[Psy-T]	Intrusive memories, fear avoidance, or PTSD symptoms
Physical	Endurance/Mobility	[Phys-E]	Functional capacity, gait, or activity level
	Motor/ROM	[Phys-M]	Specific joint movement, strength, or fine motor control
Cognitive	Delirium/Orientation	[Cog-D]	Acute confusion, disorientation, delirium, or for environmental familiarity
	Executive Function	[Cog-E]	Attention, working memory, or processing speed

**Table 8:** Axis 2—Timing of Intervention.

Category	Clinical Phase/Location	Code in Axis 2	Clinical Status (Stability: Effort Tolerance)
Pre-Acute/Prophylactic	Pre-op, elective setting	[Pre-Acute]	Stable: High Effort Tolerated
Acute/During ICU	Bedside (ICU)	[Acute]	Unstable: Minimal to Moderate Effort Only
Sub-Acute/Post-ICU	General ward, rehabilitation facility, home	[Sub-Acute]	Improving: Moderate to High Effort
Follow-up/Chronic	Post-ICU clinic, long-term care	[Chronic]	Stable/Long-term: High Effort Required

**Table 9:** Axis 3—Immersion and Interaction.

Component	Code in Axis 3	Definition and Rationale
Immersion Type	[VR]	HMD is MANDATORY. Provides stereoscopic, total field-of-view occlusion and sensory isolation for maximum presence.
	[Screen]	Low sensory isolation provided by a standard screen (e.g., tablet, computer monitor, or projection).
Interaction Modality	[Passive]	Patient is a spectator; no motor or controller input required.
	[Active]	Patient uses controllers, hand-tracking, or body movement to influence the environment (task engagement and challenge).
Content Format	[Video]	360° real-world footage or pre-rendered environments.
	[Anim]	Character- or narrative-driven animations, often used for storytelling or simple guided interventions.
	[Sim]	Detailed, interactive, or non-interactive simulation that represents an environment or scenario.
	[Game]	Rule-based games and interactive environments with defined goals and feedback loops.

**Table 10:** Axis 4—Content and Intent.

Type	Code in Axis 4	Design Intent and Level of Customization
Generic/Commercial	[GC]	Off-the-shelf, non-clinical commercial content (e.g., relaxation apps, commercial games).
Context-Specific	[CS]	Custom-made content directly relevant to the clinical context (e.g., ICU orientation, cognitive exercises).
Personalized/Adaptive	[PA]	Content that adapts in real time or is tailored to patient needs; closest to a digital therapeutic.

**Table 11:** Axis 5—Intervention Dose. Pro re nata *.

Component	Code in Axis 5	Range/Definition
Duration (gameplay)	[Brief]	5–15 min
	[Extended]	20–40+ min
	[Free]	No prescribed duration; session ends per patient or physician choice
Session Frequency	[PRN]	PRN * (as needed)
	[Single]	One session only
	[Daily]	Once or twice per day
	[Fixed]	Fixed number of sessions per week (e.g., 2–5×/week)
Intervention Period	[Short]	1–3 days
	[Medium]	4–14 days
	[Multi-Week]	≥2–4 weeks
	[Long-Term]	>4 weeks

PRN * refers to Pro re nata, meaning “as needed”.

## Data Availability

The raw data supporting the conclusions of this article will be made available by the authors on request.

## Supplementary Materials

The following supporting information can be downloaded at: https://www.mdpi.com/article/10.3390/biomedicines14020464/s1, Table S1: Full Search Strategies; Table S2: Study-level Risk of Bias Assessment (MMAT 2018); Table S3: Full-text articles excluded after eligibility assessment; Table S4: Patient Demographics and Setting Characteristics; Figure S1: The 5A-VR-PICS Classification Codes for All Included Studies.
