# Supplementary material for: Mapping the Evidence on Virtual Reality for Post-Intensive Care Syndrome: A Systematic Review and a Five-Axis VR-PICS Taxonomy
Source: Biomedicines. 2026 Feb 19;14(2):464. doi: 10.3390/biomedicines14020464 (PMC12937665; doi:10.3390/biomedicines14020464)
Supplement: Supplementary file 1 [file biomedicines-14-00464-s001.zip › biomedicines-4111756-supplementary.pdf]

Supplementary Material

Table S1. Full Search Strategies

Database	Search string	Fields/Filters applied	Date searched
Cochrane Library	(vr OR “virtual reality”) AND (icu OR “intensive care unit”)	Title, Abstract, Keywords	August 2, 2025
PubMed	(vr OR “virtual reality”) AND (icu OR “intensive care unit”)	Title/Abstract	August 2, 2025
ScienceDirect	(vr OR “virtual reality”) AND (icu OR “intensive care unit”)	Title, Abstract, Author-specified Keywords	August 2, 2025
IEEE Xplore	(vr OR “virtual reality”) AND (icu OR “intensive care unit”)	Abstract	August 2, 2025
ACM Digital Library	(vr OR “virtual reality”) AND (icu OR “intensive care unit”)	Abstract	August 2, 2025
SpringerLink	(vr OR “virtual reality”) AND (icu OR “intensive care unit”)	English language; Subjects: Virtual and augmented reality; Subdisciplines: Intensive / critical care medicine	August 2, 2025
Scopus	(vr OR “virtual reality”) AND (icu OR “intensive care unit”)	Article Title, Abstract, Keywords	August 2, 2025

Table S2. Study-level Risk of Bias Assessment (MMAT 2018)

Study	Study design	Randomization	Allocation concealment	Blinding (participants / personnel / outcome assessors)	Completeness of outcome data	Selective reporting	Overall risk of bias
Vlake et al. (2022)[9]	RCT	Adequate (1:1 randomization via Castor EDC)	Adequate (centralized sequence; investigators unaware)	No / No / Not indicated	Unclear (missing data not fully detailed)	Adequate (protocol registered)	High
Locke et al. (2024)[13]	Feasibility study	NAp	NAp	NAp	Probably adequate (no missing data indicated)	Unclear (no protocol registration)	High
Vlake et al. (2021)[10]	Case report	NAp	NAp	NAp	Adequate (complete data for single case)	Unclear (no protocol registration)	High
Ong et al. (2020)[7]	Feasibility study	NAp	NAp	NAp	Unclear (missing data not explicitly detailed)	Unclear (no protocol registration)	High
Lai et al. (2021)[14]	Case report	NAp	NAp	NAp	Adequate (complete reporting for both cases)	Unclear (no protocol registration)	High
Rousseaux et al. (2022)[12]	RCT	Adequate (computer-generated sequence)	Adequate (sealed opaque envelopes)	No / No / Yes	Adequate (ITT analysis; complete data)	Adequate (registered protocol)	Low
Badke et al. (2022)[20]	Observati onal	NAp	NAp	NAp	Adequate (all participants completed assessments)	Adequate (all outcomes reported)	High
Gerber et al. (2019) [11]	Feasibility study	NAp	NAp	NAp	Adequate (data available for completers)	Adequate (planned outcomes reported)	High
Suvajdzic et al. (2019)[16]	Feasibility study	NAp	NAp	NAp	Adequate (complete data for completers)	Adequate (preliminary outcomes reported)	High

de Vries et al. (2025)[1]	Feasibility study	NAp	NAp	NAp	Adequate (complete outcome data)	Adequate (feasibility outcomes fully reported)	High
el Mathari et al. (2024)[15]	RCT	Adequate (computer-generated, block randomization)	Adequate (centralized electronic system)	No / No / Partial	Adequate for primary; unclear for secondary outcomes	Adequate (registered protocol)	Low

Table S3. Full-text articles excluded after eligibility assessment

Author, Year	Study	Primary reason for exclusion	Criterion
Hoffman et al. (2019)[22]	Immersive virtual reality as an adjunctive non-opioid analgesic for pre-dominately Latin American children with large severe burn wounds during burn wound cleaning in the intensive care unit: a pilot study	Does not specify a VR HMD and does not target PICS-related outcomes	1; 2
ÖZBAŞ et al. (2024)[19]	The Effect of Virtual Reality Application on Pain and Anxiety During Chest Tube Removal Following Open Heart Surgery: randomized Controlled Study	Does not target PICS-related outcomes	2
Laghlam et al. (2021)[21]	Virtual reality vs. Kalinox for management of pain in intensive care unit after cardiac surgery: a randomized study.	Does not target PICS-related outcomes	2
Gomes et al. (2019)[23]	Rehabilitation through virtual reality: physical activity of patients admitted to the intensive care unit.	Does not specify a VR HMD	1
Kim et al. (2025)[24]	Effects of Virtual Reality Meditation on Sleep and Delirium in ICU Patients: A Randomized Controlled Trial.	Does not target PICS-related outcomes	2
Wang et al. (2025)[25]	Effectiveness of virtual reality assisted active limb movement exercises for patients in the respiratory intensive care unit: a randomized pilot study.	Does not specify a VR HMD	1
Groenveld et al. (2024)[26]	Pulmonary and Physical Virtual Reality Exercises for Patients With Blunt Chest Trauma: Randomized Clinical Trial.	Does not target PICS-related outcomes	2
Ahmad et al. (2024)[27]	Effects of conventional versus virtual reality-simulated treadmill exercise on fatigue, cognitive function, and participant satisfaction in post-COVID-19 subjects. A randomized trial	Does not specify a VR HMD	1
Martí-Hereu et al. (2024)[28]	Usage of immersive virtual reality as a relaxation method in an intensive care unit	Does not target PICS-related outcomes	2
Ribeiro et al. (2024)[29]	Evaluating Constrained Users Ability to Interact with Virtual Reality Applications	Does not specify a VR HMD and does not target PICS-related outcomes	1; 2
Sooriyaghandan et al. (2023)[30]	Satisfaction and tolerability using virtual reality (VR) as adjunctive treatment during flexible bronchoscopy: a randomized control trial	Does not target PICS-related outcomes	2

Twamley et al. (2024)[31]	Exploring the perceptions of former ICU patients and clinical staff on barriers and facilitators to the implementation of virtual reality exposure therapy: A qualitative study	Does not target PICS-related outcomes	2
Raya et al. (2023)[32]	Development of a Virtual Reality Tool for the Treatment of Pediatric Patients in the ICU	Insufficient outcome data	4; 5
Naef et al. (2023)[33]	Effects of immersive virtual reality on sensory overload in a random sample of critically ill patients	Does not target PICS-related outcomes	2
Haghedooren et al. (2024)[34]	Feasibility and safety of interactive virtual reality upper limb rehabilitation in patients with prolonged critical illness	Does not target PICS-related outcomes	2
Lahir et al. (2022)[35]	Effect of virtual reality experience on distress in children admitted to the intensive care unit: a randomized controlled trial	Does not target PICS-related outcomes	2
Navarra-Ventura et al. (2021)[36]	Virtual Reality-Based Early Neurocognitive Stimulation in Critically Ill Patients: A Pilot Randomized Clinical Trial	Does not specify a VR HMD	1
Jawed et al. (2021)[37]	Feasibility of a virtual reality intervention in the intensive care unit	Does not target PICS-related outcomes	2
Chillura et al. (2020)[38]	Advances in the rehabilitation of intensive care unit acquired weakness: A case report on the promising use of robotics and virtual reality coupled to physiotherapy	Does not specify a VR HMD	1
Brungardt et al. (2021)[39]	Virtual Reality-Based Music Therapy in Palliative Care: A Pilot Implementation Trial	Does not target PICS-related outcomes	2
Lee and Kang (2020)[40]	Effect of virtual reality meditation on sleep quality of intensive care unit patients: A randomised controlled trial	Does not target PICS-related outcomes	2
Blair et al. (2019)[41]	Virtual reality use in adult ICU to mitigate anxiety for a patient on V-V ECMO	Insufficient outcome data	5
Badke et al. (2019)[42]	An Innovative Virtual Reality Experience in the PICU: A Pilot Study	Does not target PICS-related outcomes	2
Gerber et al. (2017)[43]	Visuo-acoustic stimulation that helps you to relax: A virtual reality setup for patients in the intensive care unit	Does not target PICS-related outcomes	2
Ju et al. (2025)[44]	Enhancing patient experience in the surgical ICU through virtual reality: A pre-post mixed-methods study	Does not target PICS-related outcomes	2

Anderson et al. (2025)[45]	VIRTUAL REALITY SIMULATION TO IMPROVE POSTOPERATIVE CARDIOTHORACIC SURGICAL PATIENT OUTCOMES	Does not target PICS-related outcomes	2
Wu et al. (2025)[46]	Perceptions and experiences of commercial virtual reality games in early postoperative rehabilitation among cardiac surgical patients: A qualitative study	Does not target PICS-related outcomes	2
Despoti et al. (2025)[47]	Comparing virtual reality with traditional methods in cognitive rehabilitation in PICS syndrome	Does not target PICS-related outcomes	2
Peach et al. (2024)[48]	Using Compressed Virtual Reality Exposure Therapy for an Adult Critical Illness Survivor: A Case Report	Does not target PICS-related outcomes	2

Table S4. Patient Demographics and Setting Characteristics

Study	Age Range/Mean (SD/IQR)	Gender (% Male)	Setting	Design Detail
Vlake et al. (2022)[9]	62 years (SD 11)	68%	Multicenter	Prospective
Locke et al. (2024)[13]	55.4 years (SD 13.9)	55%	Multicenter	Prospective
Vlake et al. (2021)[10]	62 years	100%	Single-center	Prospective
Ong et al. (2020)[7]	68.7 years (SD 12.5)	67%	Multicenter	Prospective
Lai et al. (2021)[14]	13–15 years	100%	Single-center	Prospective
Rousseaux et al. (2022)[12]	66.8 years (SD 12)	60%	Single-center	Prospective
Badke et al. (2022)[20]	12.9 years (SD 3.9)	59%	Single-center	Cross-sectional
Gerber et al. (2019) [11]	61 years (SD 14)	57%	Single-center	Prospective
Suvajdzic et al. (2019)[16]	57.5 years (SD 15.5)	58%	Single-center	Prospective
de Vries et al. (2025)[1]	62 years (IQR 51–69)	69%	Multicenter	Prospective
el Mathari et al. (2024)[15]	64 years (SD 14.5)	60%	Single-center	Prospective

Figure S1. The 5A-VR-PICS Classification Codes for All Included Studies.

Study	A1 Therapeutic Goal	A2 Timing	A3 Immersion/Interaction	A4 Content Intent	A5 Dose
Vlake et al. (2022) [9]	Psy-A, Psy-D, Psy-T	Acute	VR/Passive/Video	CS	Brief/Single/Short
Locke et al. (2024) [13]	Psy-A	Acute	VR/Passive/Video	GC	Brief/Single
Vlake et al. (2021) [10]	Psy-A, Psy-D, Psy-T	Acute	VR/Passive/Video	CS	NR/Fixed/Medium
Ong et al. (2020) [7]	Psy-A, Psy-D; Cog-D	Acute	VR/Passive/Video/Anim	GC	Extended/Fixed/NR
Lai et al. (2021) [14]	Phys-M, Phys-E	Acute	VR/Active/Game	GC	Extended/Fixed/NR
Rousseaux et al. (2022) [12]	Psy-A	Pre-Acute/Acute	VR/Passive/Sim	CS	Extended/Fixed/NR
Badke et al. (2022) [20]	Psy-A	Acute	VR/Passive/Video	CS	Free/Single/NR
Gerber et al. (2019) [11]	Cog-D	Acute	VR/Passive/Video	CS	Brief/Fixed/Long-Term
Suvajdzic et al. (2019) [16]	Psy-A, Psy-D; Cog-D	Acute	VR/Passive/Video/Anim	GC	Free/Fixed/NR
de Vries et al. (2025) [1]	Phys-M	Acute/Sub-Acute	VR/Active/Game	PA	Extended/Fixed/Multi-Week
el Mathari et al. (2024) [15]	Psy-A	Acute	VR/Passive/Sim	CS	Extended/Fixed/NR

Study	5A Codes
Vlake et al. (2022) [9]	[Psy-A,D,T][Acute][VR/Passive/Video][CS][Brief/Single/Short]
Locke et al. (2024) [13]	[Psy-A][Acute][VR/Passive/Video][GC][Brief/Single/Short]
Vlake et al. (2021) [10]	[Psy-A,D,T][Acute][VR/Passive/Video][CS][NR/Fixed/Medium]
Ong et al. (2020) [7]	[Psy-A,D/Cog-D][Acute][VR/Passive/Video/Anim][GC][Extended/Fixed/NR]
Lai et al. (2021) [14]	[Phys-M,E][Acute][VR/Active/Game][GC]Extended/Fixed/NR]
Rousseaux et al. (2022) [12]	[Psy-A][Pre-Acute][Acute][VR/Passive/Sim][CS][Extended/Fixed/NR]
Badke et al. (2022) [20]	[Psy-A][Acute][VR/Passive/Video][CS][Free/Single/NR]
Gerber et al. (2019) [11]	[Cog-D][Acute][VR/Passive/Video][CS][Brief/Fixed/Long-Term]
Suvajdzic et al. (2019) [16]	[Psy-A,D][Cog-D][Acute][VR/Passive/Video/Anim][GC][Free/Fixed/NR]
de Vries et al. (2025) [1]	[Phys-M][Acute][Sub-Acute][VR/Active/Game][PA][Extended/Fixed/Multi-Week]
el Mathari et al. (2024) [15]	[Psy-A][Acute][VR/Passive/Sim][CS][Extended/Fixed/NR]

Reference

22. Hoffman, H.G.; Rodriguez, R.A.; Gonzalez, M.; Bernardy, M.; Peña, R.; Beck, W.; et al. Immersive virtual reality as an adjunctive non-opioid analgesic for predominantly Latin American children with large severe burn wounds during burn wound cleaning in the intensive care unit: A pilot study. Front. Hum. Neurosci. 2019, 13, 262. https://doi.org/10.3389/fnhum.2019.00262

23. Gomes, T.T.; Schujmann, D.S.; Fu, C. Rehabilitation through virtual reality: Physical activity of patients admitted to the intensive care unit. Rev. Bras. Ter. Intensiva 2019, 31, 456–463. https://doi.org/10.5935/0103-507x.20190078

24. Kim, S.; Kang, J. Effects of virtual reality meditation on sleep and delirium in ICU patients. CIN Comput. Inform. Nurs. 2025, 43. https://doi.org/10.1097/cin.0000000000001307

25. Wang, J.; Shi, C.; Jia, Y.; Xiao, Q. Effectiveness of virtual reality assisted active limb movement exercises for patients in the respiratory intensive care unit: A randomized pilot study. J. Rehabil. Med. 2025, 57, jrm28399. https://doi.org/10.2340/jrm.v57.28399

26. Groenveld, T.D.; Smits, I.G.; Scholten, N.; De Vries, M.; Van Goor, H.; Stirler, V.M. Pulmonary and physical virtual reality exercises for patients with blunt chest trauma: Randomized clinical trial. JMIR Serious Games 2024, 12, e54389. https://doi.org/10.2196/54389

27. Ahmad, A.M.; Allah, S.A.M.A.; Elhaseeb, G.A.A.; Elsharawy, D.E.; Ahmed, H.S.; Abdelwahab, M.A.M. Effects of conventional versus virtual reality-simulated treadmill exercise on fatigue, cognitive function, and participant satisfaction in post-COVID-19 subjects: A randomized trial. J. Exerc. Sci. Fit. 2024, 22, 316–321. https://doi.org/10.1016/j.jesf.2024.04.003

28. Martí-Hereu, L.; Navarra-Ventura, G.; Navas-Pérez, A.M.; Fernández-Gonzalo, S.; Pérez-López, F.; De Haro-López, C.; et al. Usage of immersive virtual reality as a relaxation method in an intensive care unit. Enferm. Intensiva 2023, 35, 107–113. https://doi.org/10.1016/j.enfie.2023.08.005

29. Ribeiro, T.; Henriques, P.R.; Oliveira, E.; Rodrigues, N.F. Evaluating constrained users ability to interact with virtual reality applications. In Proc. IEEE 12th Int. Conf. Serious Games Appl. Health (SeGAH); IEEE: 2024; pp. 1–8. https://doi.org/10.1109/segah61285.2024.10639581

30. Sooriyaghandan, I.V.; Jailaini, M.F.M.; Abeed, N.N.N.; Ng, B.H.; Yu-Lin, A.B.; Shah, S.A.; et al. Satisfaction and tolerability using virtual reality as adjunctive treatment during flexible bronchoscopy: A randomized control trial. BMC Pulm. Med. 2023, 23, 10. https://doi.org/10.1186/s12890-023-02304-y

31. Twamley, J.; Hamer, O.; Hill, J.; Kenyon, R.; Twamley, H.; Casey, R.; et al. Exploring the perceptions of former ICU patients and clinical staff on barriers and facilitators to the implementation of virtual reality exposure therapy: A qualitative study. Nurs. Crit. Care 2022, 29, 313–324. https://doi.org/10.1111/nicc.12868

32. Raya, L.; Ruiz, J.J.; Fabián, M.; Ron, A.; García, J.; Verdú, C. Development of a virtual reality tool for the treatment of pediatric patients in the ICU. IEEE Comput. Graph. Appl. 2023, 43, 69–77. https://doi.org/10.1109/mcg.2023.3239676

33. Naef, A.C.; Gerber, S.M.; Single, M.; Müri, R.M.; Haenggi, M.; Jakob, S.M.; et al. Effects of immersive virtual reality on sensory overload in a random sample of critically ill patients. Front. Med. 2023, 10, 1268659. https://doi.org/10.3389/fmed.2023.1268659

34. Haghedooren, E.; Haghedooren, R.; Langer, D.; Gosselink, R. Feasibility and safety of interactive virtual reality upper limb rehabilitation in patients with prolonged critical illness. Aust. Crit. Care 2024, 37, 949–956. https://doi.org/10.1016/j.aucc.2024.06.004

35. Lahiri, R.; Metgud, D. Effect of virtual reality experience on distress in children admitted to the intensive care unit: A randomized controlled trial. Sri Lanka J. Child Health 2022, 51, 572–577. https://doi.org/10.4038/sljch.v51i4.10372

36. Navarra-Ventura, G.; Gomà, G.; De Haro, C.; Jodar, M.; Sarlabous, L.; Hernando, D.; et al. Virtual reality-based early neurocognitive stimulation in critically ill patients: A pilot randomized clinical trial. J. Pers. Med. 2021, 11, 1260. https://doi.org/10.3390/jpm11121260

37. Jawed, Y.T.; Golovyan, D.; Lopez, D.; Khan, S.H.; Wang, S.; Freund, C.; et al. Feasibility of a virtual reality intervention in the intensive care unit. Heart Lung 2021, 50, 748–753. https://doi.org/10.1016/j.hrtlng.2021.05.007

38. Chillura, A.; Bramanti, A.; Tartamella, F.; Pisano, M.F.; Clemente, E.; Lo Scrudato, M.; et al. Advances in the rehabilitation of intensive care unit acquired weakness. Medicine (Baltimore) 2020, 99, e20939. https://doi.org/10.1097/md.0000000000020939

39. Brungardt, A.; Wibben, A.; Tompkins, A.F.; Shanbhag, P.; Coats, H.; LaGasse, A.B.; et al. Virtual reality-based music therapy in palliative care: A pilot implementation trial. J. Palliat. Med. 2020, 24, 736–742. https://doi.org/10.1089/jpm.2020.0403

40. Lee, S.Y.; Kang, J. Effect of virtual reality meditation on sleep quality of intensive care unit patients: A randomised controlled trial. Intensive Crit. Care Nurs. 2020, 59, 102849. https://doi.org/10.1016/j.iccn.2020.102849

41. Blair, G.J.; Kapil, S.; Cole, S.P.; Rodriguez, S. Virtual reality use in adult ICU to mitigate anxiety for a patient on V-V ECMO. J. Clin. Anesth. 2018, 55, 26–27. https://doi.org/10.1016/j.jclinane.2018.12.033

42. Badke, C.M.; Essner, B.S.; O'Connell, M.; Malakooti, M.R. An innovative virtual reality experience in the PICU: A pilot study. Pediatr. Crit. Care Med. 2019, 20, e283–e286. https://doi.org/10.1097/pcc.0000000000001917

43. Gerber, S.M.; Jeitziner, M.M.; Wyss, P.; Chesham, A.; Urwyler, P.; Müri, R.M.; et al. Visuo-acoustic stimulation that helps you to relax: A virtual reality setup for patients in the intensive care unit. Sci. Rep. 2017, 7, 13228. https://doi.org/10.1038/s41598-017-13153-1

44. Ju, X.; Jiang, L.; Yang, J.; Zheng, Q.; Liu, X. Enhancing patient experience in the surgical ICU through virtual reality: A pre-post mixed-methods study. Heart Lung 2024, 70, 93–101. https://doi.org/10.1016/j.hrtlng.2024.11.014

45. Anderson, R.J.; Bauer, P.R.; Arghami, A.; Haney, R.M.; Reisdorf, E.M.; Baalson, K. Virtual reality simulation to improve postoperative cardiothoracic surgical patient outcomes. Am. J. Crit. Care 2025, 34, 111–118. https://doi.org/10.4037/ajcc2025704

46. Wu, Z.; Li, X.; Li, L.; Zhang, Y.; Shen, X. Perceptions and experiences of commercial virtual reality games in early postoperative rehabilitation among cardiac surgical patients: A qualitative study. Digit. Health 2025, 11, 20552076251315793. https://doi.org/10.1177/20552076251315793

47. Despoti, A.; Patsaki, I.; Alexandropoulou, A.; Magkouti, E.; Tzoumi, D.; Leventakis, N.; et al. Comparing virtual reality with traditional methods in cognitive rehabilitation in PICS syndrome. Appl. Neuropsychol. Adult 2025, 1–12. https://doi.org/10.1080/23279095.2025.2477184

48. Peach, B.C.; Cox, C.L. Using compressed virtual reality exposure therapy for an adult critical illness survivor: A case report. Crit. Care Nurse 2024, 44, 35–40. https://doi.org/10.4037/ccn2024513
